# Assessing the Educational Needs of Family Caregivers of Psychiatric Patients in Jeddah, Saudi Arabia

**DOI:** 10.7759/cureus.53364

**Published:** 2024-02-01

**Authors:** Amani Sabban, Hanaa Y Shahin, Rehab Hawsawi, Adnan Almohammadi, Afnan Aboyunis, Wafaa Alshehri, Shada Alahmadi, Rajwa Awadd, Roua Alsharif, Dhaifallah Albqomi, Abdullah Almutayri, Musleh Alharbi

**Affiliations:** 1 Transformation, King Fahad General Hospital, Jeddah, SAU; 2 Paramedical Science, King Fahad General Hospital, Jeddah, SAU; 3 Physiotherapy, King Fahad General Hospital, Jeddah, SAU; 4 Nursing, Rabigh General Hospital, Rabigh, SAU; 5 Medicine and Surgery, East Jeddah General Hospital, Jeddah, SAU; 6 Family Medicine, King Fahad General Hospital, Jeddah, SAU; 7 Nursing, East Jeddah General Hospital, Jeddah, SAU; 8 Public Health, Ministry of Health, Jeddah, SAU; 9 Public Health, Vaccination Center, King Abdulaziz Airport, Jeddah, SAU

**Keywords:** caregiver challenges, family caregiver, professional caregivers, caregivers' burden, psychiatric nursing, psychiatric patient, education needs assessment

## Abstract

Background

Family caregivers of psychiatric patients in Saudi Arabia and most of the Eastern world are suffering a big burden as a result of their caregiving role.

Aim

This study aims to assess the need for psychoeducation for family caregivers of psychiatric patients from outpatient clinics in a psychiatric hospital in Jeddah, Saudi Arabia.

Materials and methods

A cross-sectional descriptive-analytical study was conducted, which included a total of 379 family caregivers providing care to a patient suffering from a psychiatric illness.

Results

The majority of family caregivers looking after a patient suffering from a psychiatric illness were a brother or sister (20.8%), followed by a son or daughter (20.6%), and lastly, a spouse (10.3%). The top 10 important educational needs of the caregivers included their desire to know about the daily treatment of the patient, followed by how to improve social relationships, and a need for effective stress management. Family caregivers also showed interest in stress and illness. The caregivers' areas of least concern were about the admission of the patients to psychiatric hospitals, recent research on mental illnesses, and how to deal with weight gain.

Conclusion

The study showed that caregivers demonstrated a need for more experience in how to care for psychiatric patients. Indeed, this has an interrelated impact on the general well-being of both the patient and the caregiver. The study recommends the need for care guidelines to be provided by hospitals from the patient’s education department for caregiving to help the family in their daily care.

## Introduction

A family member commonly provides the care of psychiatric patients. The family caregiver usually suffers a big burden due to insufficient or insufficient knowledge. Knowledge of effective and proper caregiving, suitable interventions, overcoming stress, reducing the burden, and coping with the disorder is important. Therefore, professional and ongoing support from the mental health team may benefit family caregivers.

Limited support is available in the community for patients suffering from psychiatric disorders and their families in Middle Eastern countries. An ambivalent attitude from society towards psychiatric patients is often experienced. Instead of assisting the psychiatric patient, neighbors often avoid interaction with mentally ill patients and their families [1]. Thus, there is a need for psychoeducational programs to provide knowledge, develop essential understanding, and show more acceptability and compliance in coping and involving the family during the patient’s intervention [2].

The experience of the burden of a family caregiver is greatly influenced by their cognitive appraisal of the situation and their psychosocial skills for coping with it. Research findings suggest that information and support provided by psychiatrists and other professionals can alleviate the burden on caregivers [3]. These caregivers often experience a continuum of reactions to caring for patients after hospitalization. Some caregivers report few disruptions, while others report multiple impacts, including upsetting daily activities and stigma.

Caregivers may experience stigma associated with patients’ psychiatric hospital visits; this stigma may be reflected in caregivers’ negative self-evaluation, feelings of shame or embarrassment, or perceptions of being viewed or treated differently by others, apparently because of their caretaking role and association with the patient [3]. Caregivers may also be self-devaluing or concerned about others’ perceptions about the causes of the illness.

For nearly three decades, research on the effects of caregiving on people with psychiatric diseases has been limited. Little attention has been paid to assessing the dimensions of caregiving for patients with different psychiatric illnesses with respect to the family caregiver’s burden and coping strategies. Previous studies have reported that family caregivers commonly change their sleeping and eating habits [4], have disruptions in work and everyday activities [5], and are prone to violence and suicide attempts [6].

Poor health education plays a crucial role in the care process, and it is considered a risk factor for psychiatric illnesses [7, 8]. Education has a strong element of empowerment and should be considered in psychiatric prevention and mental health promotion. Family caregivers should be assessed and provided with sufficient and required psychoeducation.

Psychoeducation may include a possible reaction to hospitalization or scheduled follow-up. Also, caregivers should be taught coping skills that may improve clinical outcomes for the patient. This was supported previously by a study that assessed the need for education for psychiatric patients’ family caregivers, as they play an active role in the treatment of psychiatric illnesses [7].

Educating family caregivers helps decrease the relapse rate and the residual symptoms of the psychiatric disorder. Additionally, it enhances the family caregiver’s communication with society [8].

Family caregivers have been reporting escalating desires for more psychoeducation and continuous communication with mental health professionals. It has been reported that psychoeducation can make them feel less burdened, more effective in helping their loved ones, have fewer psychosomatic symptoms and burnout, and experience fewer levels of distress [9,10,3,11].

There is strong evidence suggesting that fewer families of patients with schizophrenia receive outpatient services as few facilities in the Veterans Affairs healthcare system have offered evidence-based psychoeducational programs to families [12].

According to Mental Health America (MHA) and the National Alliance on Mental Illness (NAMI), family caregivers spend 32 hours a week assisting their loved ones on average, and nearly one in five spend more than 40 hours per week [13]. Family caregivers expressed concerns about the quality and level of access to psychiatric care for their loved ones. More than half were dissatisfied with the amount and type of community services provided to their loved ones. Family caregivers in rural areas expressed more difficulties with access and quality; they complained of providing more hours of care than their counterparts in urban/suburban areas.

Rationale

The current understanding of the healthcare system is rapidly changing, which suggests that family caregivers need to be re-assessed regarding relevance and new insights in response to psychiatric illness. Limited studies have assessed the area of patient and community awareness. This makes it difficult to assess the needs of the family caregivers, the family’s knowledge, responses, and management of mental illness.

Aim of the study

Therefore, the aim of the study is to assess the psychoeducational needs of family caregivers of psychiatric patients from outpatient clinics in psychiatric hospitals in Jeddah, Saudi Arabia. This cross-sectional study was conducted to examine the relationship between related issues of interest as they exist in the population for a short period of time.

## Materials and methods

Study design

A cross-sectional study included a total of 379 caregivers of psychiatric patients. The study assessed the burden of caregivers providing care for patients at home from January to May 2017. All study participants were living in Jeddah, Saudi Arabia. All study participants met the study inclusion criteria which were as follows: first, study participants must be Saudi citizens aged 18 or older; second, the participant must be a relative caregiver and have lived with the patient for the last 12 months; third, all participants must accompany a patient who is diagnosed with a psychiatric disease, had a regular follow-up appointment at the outpatient clinic, and was not suffering from mental illness. The study participants were randomly selected from a hospital computer list at an outpatient clinic. A total of 383 participants among the 79,000 registered patients completed the survey. Among those, four respondents were excluded from the study as the questionnaire was incomplete. Therefore, the total study sample was 379 people who completed the survey. This study was approved by the Institutional Review Board (IRB) of the Directorate of Health Affairs, Saudi Arabia (IRB approval number: IRB-A-00478). Written consent was obtained from each respondent in the outpatient department.

Study instrument/questionnaire

The questionnaire was presented in the English language and consisted of two parts. The first part contained general information about the sample. The second part was adapted from the Educational Needs Questionnaire (AM-ENQ) of Kim T. Mueser [14] (Appendix A). Permission to reproduce the questionnaire has been obtained. The scale to assess the psychoeducational needs of psychiatric patients and their caregivers contained 45 items related to different areas of psychoeducational needs for psychiatric patients and family caregivers.

The questionnaire was rated on a five-point Likert scale, ranging from one (not important) to five (very important). The questionnaire consisted of six domains. The first domain covered the basic facts of dealing with mental illness (13 items). The second domain was about coping with patients’ symptoms (11 items). The third domain covered social functioning enhancement (six items). The fourth domain covered community resources (six items). The fifth domain covered family problems in coping with stress (six items). The last domain consisted of miscellaneous items, such as dealing with weight gain (three items).

Translation of the questionnaire was conducted using the following steps: the first step was translating the original English questionnaire into Arabic by two independent translators working at the Jeddah Psychiatric Hospital in Jeddah, Saudi Arabia. Second, the two translators then met to discuss the translated versions of the questionnaire and agreed on one synthesized Arabic version. Third, the synthesized Arabic version was then translated back into English by two independent translators. Fourth, the updated version was compared with the original questionnaire by a committee of experts who determined the updated questionnaire was identical to the original questionnaire in the English language. This was then sent to the original author, who approved the final version. Fifth, the face validity of the Arabic version of the questionnaire was tested by an expert in the medical field. Slight modifications were suggested, such as re-wording for clarity in Arabic. Finally, the English version was administered to 10 members of the hospital staff. After 15 days, the Arabic version was administered to the same members, and the results were compared. To check the validity, we tested the questionnaire using Cronbach's alpha method.

Pilot study

The AM-ENQ took 20 minutes to be completed, and 35 family caregivers were assessed. Family caregivers were randomly selected from the outpatient clinic and emergency room in Jeddah Psychiatric Hospital, Jeddah, Saudi Arabia. As a result, the questionnaire was considered easy to complete, with no further modifications required.

Data collection and analysis

Data collection was carried out by the researcher during working hours in April 2017. After the introduction, the researcher explained the aim of the study and provided advice on how to answer the questionnaire. Each respondent completed a self-administered questionnaire, which was handed to the researcher. The data were analyzed using IBM SPSS Statistics software version 21 for Windows (IBM Corp., Armonk, NY). Both descriptive and inferential statistics were used to present the results. A p-value less than or equal to 0.05 is considered significant.

## Results

The total number of people who completed the survey was the study's sample, and the response rate was 98%. The results covered two main sections: the first one described the characteristics of the respondents, and the second section presented the mean scores for each of the items included in the AM-ENQ, together with a comparison of the responses according to the characteristics of the patients and their caregivers.

To check the validity of the questionnaire, we used Cronbach's alpha method. Table 1 shows the results of Cronbach's alpha for each domain.

**Table 1:** Reliability of the domain and overall items of the Educational Need Questionnaire (AM-ENQ) (r >0.985).

Domains	Cronbach's alpha
Basic facts about mental illness (13 items)	0.953
Coping with patient symptoms (11 items)	0.960
Enhancing social functioning (6 items)	0.926
Community resources (6 items)	0.914
Coping with stress and family problems (6 items)	0.918
Miscellaneous (3 items)	0.859
Overall	0.985

Overall, the Cronbach's alpha test scores for all domains were above 0.9. The overall score was 0.985, demonstrating the acceptable validity of the questionnaire.

The demographic data were analyzed for 379 caregivers and is shown in Table 2.

**Table 2:** Demographic characteristics of the caregivers (n=379) The table demonstrates the differences in the overall educational need score in relation to the characteristics of the caregivers. A p-value less than or equal to 0.05 is considered significant. SR: Saudi riyal

Characteristics	Frequency (%)	Mean (SD)	p-value
Gender			
Male	157 (41.4)	3.40 (1.02)	0.098*
Female	222 (58.6)	3.58 (1.00)	
Age: mean (range)			
<30 years	178 (47.0)	3.65 (0.96)	0.033**
30-<40 years	130 (34.3)	3.41 (1.06)	
≥ 40 years	71 (18.7)	3.32 (1.01)	
Marital status			
Single	158 (41.7)	3.48 (0.99)	0.424**
Married	164 (43.3)	3.48 (1.03)	
Divorced	45 (11.9)	3.73 (0.93)	
Widowed	12 (3.2)	3.30 (1.27)	
Education level			
Illiterate	25 (6.6)	3.29 (0.54)	<0.001**
Primary	16 (4.2)	2.99 (1.24)	
Intermediate	42 (11.1)	3.39 (0.91)	
Secondary	61 (16.1)	3.30 (1.09)	
University	211 (55.7)	3.71 (0.92)	
Postgraduate	24 (6.3)	3.44 (1.40)	
Income			
<5,000 SR	115 (30.3)	3.18 (0.95)	<0.001**
5,000-10,000 SR	209 (55.1)	3.66 (0.95)	
>10,000 SR	55 (14.5)	3.57 (1.21)	
* Based on an Independent sample t-test; ** Based on ANOVA test			

Overall, there was no significant difference between gender, age, and marital status of caregivers. The mean age group of caregivers was 32.2 years, ranging from 15 to 70 years old. Our data showed approximately 47% (n=178) of the caregivers were less than 30 years old, and the smallest percentage were 40 years and older (18.7%; n=71). The mean age was 32.2 years (SD±9.2). Additionally, 43.3% (n=164) of caregivers were married, followed by 41.7% (n=158) who were single, and the rest were either divorced (11.9%; n=45) or widowed (3.2%; n=12). More than 55% (n=211) of the caregivers had completed an undergraduate degree from the university. Table 2 revealed that 209 (55.1%) of the caregivers had a monthly income ranging between 5,000 SR and 10,000 SR (Saudi riyal).

Moreover, Table 2 demonstrates the differences in the overall educational need score in relation to the characteristics of the caregivers. Our results show no statistical difference between males and females in terms of higher educational needs (3.40±1.02 and 3.58 ±1.00, respectively).

Additionally, caregivers who had university qualifications indicated significantly higher scores for the importance of educational needs (3.71±0.92), when compared with caregivers with lower educational levels (p<0.05). Accordingly, the high educational needs score was significantly reported by caregivers who had monthly income >10,000 SR (3.57±1.21) and monthly income between 5,000 SR and 10,000 SR (3.66±0.95) (p<0.05). Generally, there was no significant difference in the importance of educational needs according to the caregiver's marital status.

**Figure 1:**
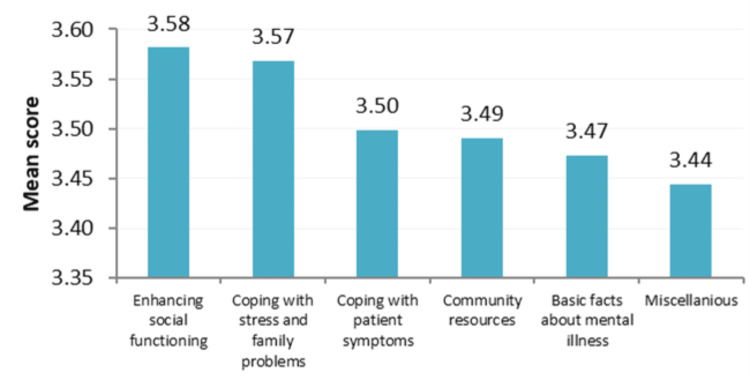
Mean scores (x-axis) for the dimensions of the educational needs of the caregivers (y-axis).

Figure 1 demonstrates the mean scores for the dimensions of the educational needs of the caregivers.

The highest educational needs of the participants were centered on topics like enhancing social functioning (mean=3.58), followed by family coping with stress (mean=3.57). The topics with the lowest concerns of the caregivers were directed towards the basic facts of the mental illness (mean=3.47) and miscellaneous items (mean=3.44), which were weight gain, the death of one of the parents, coping with holidays, and how to deal with stigma.

Table 3 demonstrates the reported diagnosis of the patients and their relationship with the caregivers.

**Table 3:** The differences in the overall educational need score according to the characteristics of the patients and their relationships with the caregivers A p-value less than or equal to 0.05 is considered significant.

Characteristics	No. (%)	Mean (SD)	p-value
Reported diagnosis			
Schizophrenia	48 (12.7)	3.66 (0.85)	0.065**
Depression	174 (45.9)	3.54 (1.03)	
Schizoaffective	48 (12.7)	3.55 (1.04)	
Mood disorder	57 (15.0)	3.56 (0.93)	
Others	52 (13.7)	3.10 (1.09)	
Duration since starting treatment			
<1 year	104 (27.4)	3.21 (1.15)	<0.001**
1-2 years	122 (32.2)	3.49 (0.81)	
>2 years	153 (40.4)	3.75 (1.02)	
Relation between the respondent caregiver and the patient			
Son/daughter	78 (20.6)	3.81 (0.81)	<0.001**
Parent	49 (12.9)	3.49 (0.98)	
Spouse	39 (10.3)	3.78 (0.82)	
Brother/sister	79 (20.8)	3.55 (0.95)	
Friend	63 (16.6)	3.29 (1.18)	
Relative	71 (18.7)	3.14 (1.11)	
The caregiver shares direct constant care for the patients			
Yes	324 (85.5)	3.54 (1.01)	0.114*
No	55 (14.5)	3.30 (1.02)	
Duration of providing care (n=324)			
<4 years	182 (56.2)	3.50 (1.01)	0.007**
4≤6 years	75 (23.1)	3.37 (1.02)	
6-8 years	56 (17.3)	3.72 (0.94)	
>8 years	11 (3.4)	4.39 (0.58)	
Time of daily care			
<4 hours	150 (46.3)	3.36 (1.06)	<0.001**
4≤6 hours	91 (28.1)	3.48 (0.99)	
6-8 hours	71 (21.9)	3.81 (0.81)	
>8 hours	12 (3.7)	4.51 (0.56)	
* Based on an Independent sample t-test; ** Based on ANOVA test			

Among other diagnoses, the most commonly identified diagnosis for patients was depression (45.9%; n=174). The other diagnosis included manic disorder (13.7%; n=52).

All patients had already started treatment at the time of the study. Approximately 40% of these patients had been on treatment for more than two years. Approximately 32.2% were on treatment for at least one year.

In terms of the relationship between the caregivers and the patients, the majority of caregivers were closely related to the patients. The caregiver was a son or daughter (20.6%; n=78), parent of the patient (12.9%; n=49), a spouse (10.3%; n=39), brother or sister (20.8%; n=79), or any other relative (18.7%; n=71). Approximately 16.6% (n=63) of the caregivers were friends of the patients.

A majority of the respondents (85.5%; n=324) indicated that they provided close care to the patient. Among those, 56.2% (n=182) of caregivers reported that they had been providing care for less than four years. Only a few caregivers (3.4%; n=11) had been providing care for more than eight years. In terms of the number of hours per day, about 46.3% (n=150) of caregivers provide less than four hours of daily care for the patient.

Table 3 also indicates that the longer the duration of the treatment, the higher the necessity of the educational needs for the caregivers. The mean score increased significantly for those who are providing care for patients with a duration of treatment of less than one year and those with a duration of treatment of more than two years (3.21±1.15 and 3.75±1.02, respectively) (p<0.05).

The mean score for educational need was significantly higher amongst sons or daughters providing care for a patient (3.81±0.81), and the lowest score was recorded in the other distant relatives (3.41±1.11) (p<0.05).

The mean score increased significantly with the increased duration of care. It reached up to (4.39±0.58) in those who had been providing care for more than eight years. Moreover, educational needs increased with an increased number of hours spent on daily care. Caregivers who were providing daily care for more than eight hours had more educational needs than those who were providing daily care for less than four hours (4.51±0.56 and 3.36±1.06, respectively) (p<0.05). Consistent with previous studies, our results showed that the longer the duration of treatment, the higher the necessity of the educational needs for the caregivers.

Table 4 illustrates the mean and standard deviation for the top and lowest 10 educational needs of the caregivers.

**Table 4:** The educational needs of the caregivers according to their responses (Educational Needs Questionnaire (AM-ENQ)). The highest and the lowest educational needs are presented.

Educational needs	Mean	SD
The top 10 educational needs		
Day treatment	3.74	1.30
Improving social relationships	3.72	1.28
Ways of managing stress more effectively	3.70	1.26
Stress and illness	3.65	1.27
Improving communication with relatives	3.65	1.30
Strategies for solving problems	3.63	1.27
Early warning signs of the illness and relapse	3.61	1.25
Side effects of medications	3.60	1.27
Setting limits on the patient's behavior	3.59	1.29
Enhancing leisure and recreational activities	3.59	1.26
The least 10 educational needs:		
Drug/alcohol abuse	3.44	1.31
Involuntary commitment to the hospital	3.44	1.30
Planning/coping with holidays	3.44	1.33
Biological theories	3.44	1.31
Alternative treatment approaches	3.41	1.33
Vocational rehabilitation	3.39	1.33
Alternative living situations	3.39	1.23
Dealing with weight gain	3.38	1.31
Recent research on mental illness	3.30	1.36
Admission to psychiatric hospital	3.26	1.38

A maximum score of five or higher indicates higher importance, and vice versa. The top 10 important educational needs of the caregivers included their desire to know about the day treatment of the patient (3.74±1.30), followed by how to improve social relationships (3.72±1.28), and how to manage stress more effectively (3.70±1.26). Conversely, the areas that the caregivers were least concerned about were the admission of patients in psychiatric hospitals (3.26±1.38), recent research on mental illnesses (3.30±1.36), and how to deal with weight gain (3.38±1.31).

## Discussion

The aim of this study was to assess the educational needs of caregivers of patients with psychiatric diseases at an outpatient clinic in a psychiatric hospital in Jeddah. A total of 379 caregivers completed the survey, and over 99% of the respondents completed the AM-ENQ part. The study revealed that caregivers were interested in gaining more knowledge about psychiatric illnesses and different management strategies. Conversely, admission of patients to psychiatric hospitals and drug abuse for mental illnesses were topics of the least concern to the caregivers. This has an impact on compliance and other important needs in education programs to be offered, such as dietary advice, regular exercise, and behavior modification.

Previous studies revealed the benefits of psychoeducation. This raises awareness about psychiatric illnesses as well as helps reduce burden and distress and means to enhance coping abilities amongst caregivers. In line with this, the result of our study showed that relatives were interested in gaining more knowledge about mental illnesses and different management strategies. This is consistent with previous surveys assessing the educational needs of family caregivers [15].

Based on the six main domains of educational needs, our results showed that the highest educational needs of the participants were centered on enhancing social functioning, followed by family coping with stress. Family caregivers of psychiatric patients who participated in psychoeducational interventions showed greater improvements in their social functioning, as supported by the findings of the study by Tarrier and Barrowclough [16].

Regarding how to cope with patient symptoms, findings were consistent with those of the self-efficacy scale for coping skills needed by the family caregivers of persons with psychiatric diseases [15]. On the other hand, the least concerns of the caregivers were directed towards the basic facts of the mental illness (mean=3.47) and miscellaneous items (mean=3.44) that indicate weight gain, death of one of the parents, coping with holidays, and how to deal with stigma. The result is supported by the findings of a study by Norman et al. [17].

Regarding the relationship between the family caregivers and the patients, in this study, we showed that the majority of the caregivers were closely related to the patients. This result indicates the very close relationships of the Saudi community, which feels a responsibility towards the patient. This result is supported by Winefield and Harvey's study, which found that the majority of the respondents (85.5%) indicated that they provide close care to the patients [18].

This study revealed that more than half of the participants (56.2%; n=182) reported they were providing care for the patients for less than four years, and only 3.4% (n=11) were providing care for more than eight years. The result also shows that 46.3% (n=150) provide care for the patients for less than four hours daily; a higher burden was associated with more hours of contact with the patients.

Family caregivers of patients with psychiatric disorders who lack psychoeducation and guidance indicated that they actively seek help for knowledge, information, and skills from the specialized psychiatric team. The hospital's role, in the researcher’s opinion, is to provide guidelines through intensive educational awareness programs in addition to written information, as shown in Table 4, which shows the top 10 educational needs. This indicates the need for family caregivers for day treatment (mean=3.74), the need for improvement of social relationships (mean=3.72), the need for ways to manage stress (mean=3.70), and lastly, ways to enhance recreational activities. This result is in complete agreement with the study by Tarrier and Barrowclough [16], except for the need to enhance recreational activities. In Chien’s study, this need comes with less than 10 needs.

Educational needs vary greatly among family caregivers and often depend on the existing family condition and diagnosis. Family caregivers who have a longer history of illness may have already gained skills and taken significant steps to retrieve knowledge to support their relatives.

These results suggest that family caregivers’ psychoeducational needs cannot be adequately addressed by simply providing information once. Rather, an ongoing need for provision and support is required in psychoeducation for caregivers. The psychiatric condition and status of the ill relative might benefit from a more structured behavioral program, like family psychoeducation [19].

The present study provides an outline of the educational needs of the caregivers of psychiatric patients and suggests how these needs should be met so that the outcome confirms the most significant needs among family caregivers. The results are supported by the results of the study by Johnson [20].

Although the current study provided insight for future studies in Saudi Arabia, this qualitative study was conducted in a single center at the Jeddah Psychiatric Hospital in Saudi Arabia. Thus limiting the generalizability of our findings. Future large-scale studies, including many psychiatric centers in Saudi Arabia, are required.

Our recommendation is to design and implement psychoeducational programs based on objective data collected from specific subject groups, ensuring content and structure are tailored to the individual needs and preferences of each family and therefore match their interests.

In summary, the caregiving of psychiatric patients gives rise to negative and positive experiences, and these experiences are interrelated. These experiences are directly affected by the well-being of the psychiatric patient and, in turn, affect the general well-being of caregivers. Family caregivers need to be provided with sufficient knowledge and ongoing support to help them through the grieving process, to develop positive thinking, and to accept their situation. It appears that medical education curriculum guidelines have insufficient content about family caregivers of psychiatric patients. The educational experiences of mental health professionals may not adequately prepare them for working with the family caregivers of their patients.

## Conclusions

In conclusion, this cross-sectional study highlights the need for psychoeducation for caregivers. It is recommended that medical education curriculum guidelines incorporate information about family stigma, family/caregiver burden, information exchange, family stress, coping and adaptation, family support, crisis response, and psychoeducation of multiple family groups.

## Figures and Tables

**Figure 2:**
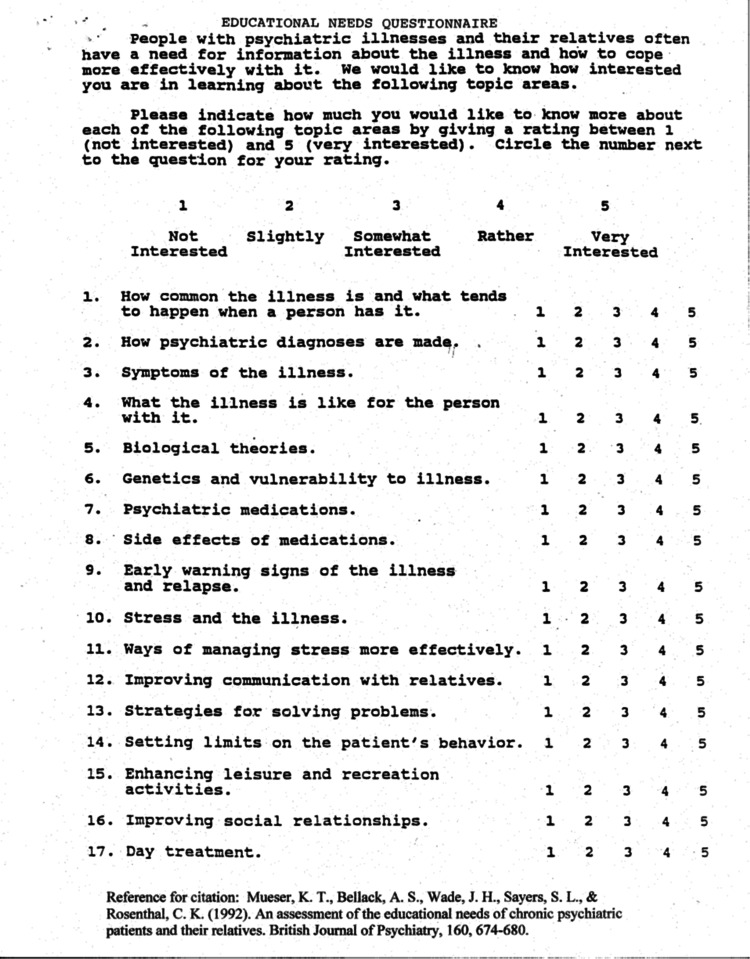
Educational Needs Questionnaire (AM-ENQ) of Kim T. Mueser [14] Permission has been obtained for the use of this questionnaire.

**Figure 3:**
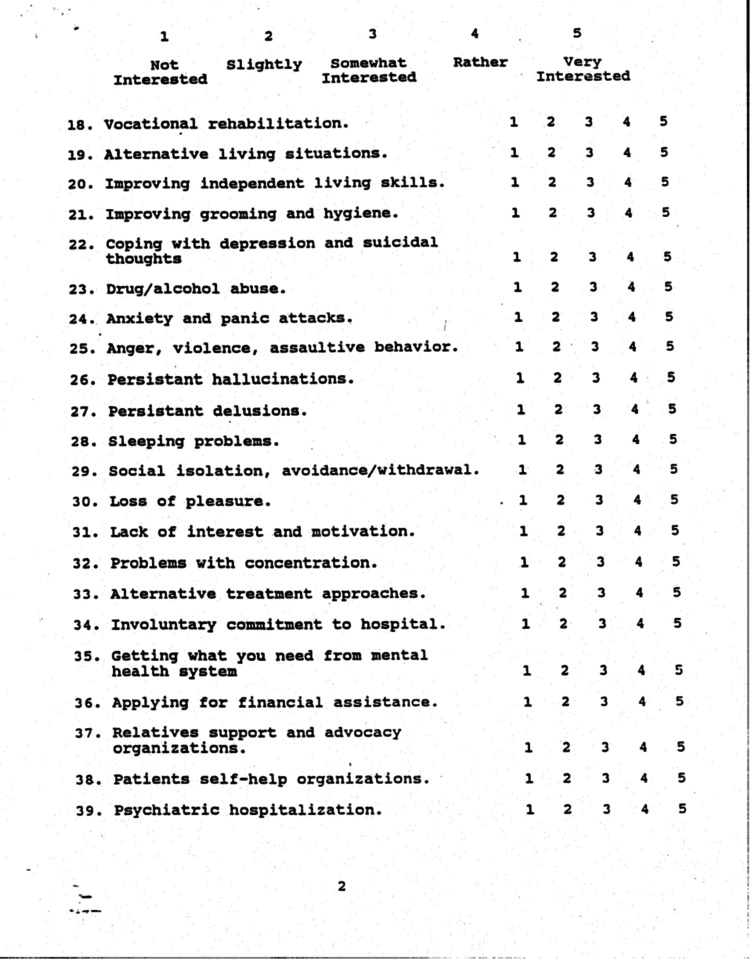
Educational Needs Questionnaire (AM-ENQ) of Kim T. Mueser [14] Permission has been obtained for the use of this questionnaire.

**Figure 4:**
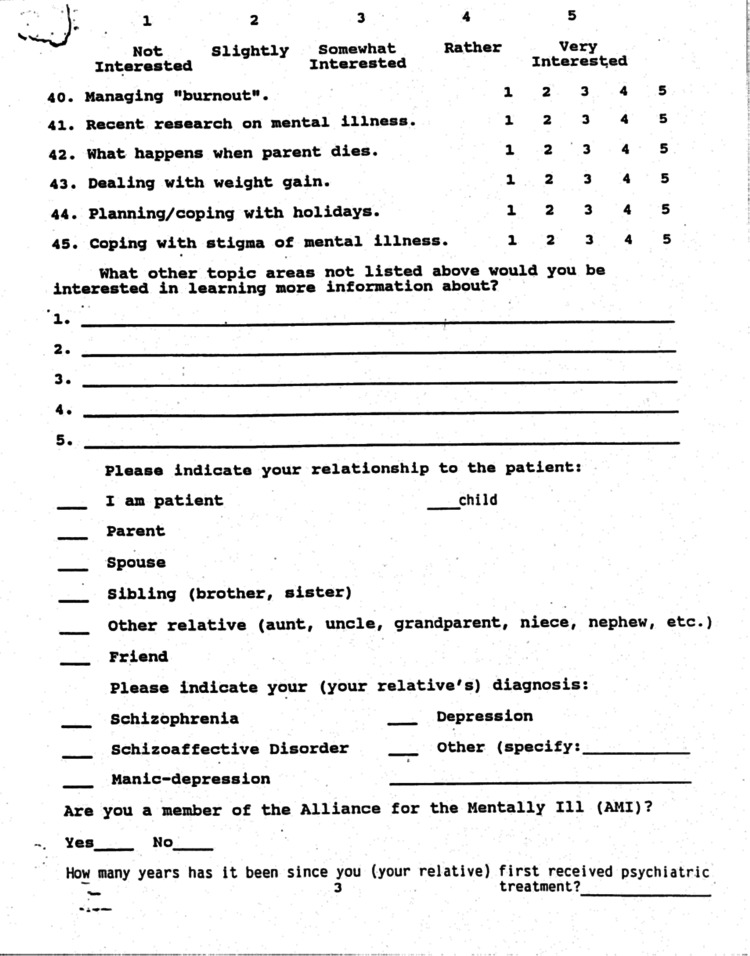
Educational Needs Questionnaire (AM-ENQ) of Kim T. Mueser [14] Permission has been obtained for the use of this questionnaire.

## Appendices

Appendix A 
